# Nucleation Promoting Factors Regulate the Expression and Localization of Arp2/3 Complex during Meiosis of Mouse Oocytes

**DOI:** 10.1371/journal.pone.0052277

**Published:** 2012-12-18

**Authors:** Jun Liu, Qiao-Chu Wang, Fei Wang, Xing Duan, Xiao-Xin Dai, Teng Wang, Hong-Lin Liu, Xiang-Shun Cui, Nam-Hyung Kim, Shao-Chen Sun

**Affiliations:** 1 College of Animal Science and Technology, Nanjing Agricultural University, Nanjing, China; 2 Department of Animal Sciences, Chungbuk National University, Cheongju, Chungbuk, Korea; Institute of Zoology, Chinese Academy of Sciences, China

## Abstract

The actin nucleation factor Arp2/3 complex is a main regulator of actin assembly and is involved in multiple processes like cell migration and adhesion, endocytosis, and the establishment of cell polarity in mitosis. Our previous work showed that the Arp2/3 complex was involved in the actin-mediated mammalian oocyte asymmetric division. However, the regulatory mechanisms and signaling pathway of Arp2/3 complex in meiosis is still unclear. In the present work, we identified that the nucleation promoting factors (NPFs) JMY and WAVE2 were necessary for the expression and localization of Arp2/3 complex in mouse oocytes. RNAi of both caused the degradation of actin cap intensity, indicating the roles of NPFs in the formation of actin cap. Moreover, JMY and WAVE2 RNAi decreased the expression of ARP2, a key component of Arp2/3 complex. However, knock down of Arp2/3 complex by Arpc2 and Arpc3 siRNA microinjection did not affect the expression and localization of JMY and WAVE2. Our results indicate that the NPFs, JMY and WAVE2, are upstream regulators of Arp2/3 complex in mammalian oocyte asymmetric division.

## Introduction

During mammalian oocyte meiotic maturation, a process called oocyte polarization occurs, which leads to a unique asymmetric division. The primary oocyte generates two daughter cells, a highly polarized big metaphase II (MII)-arrested oocyte and a small polar body, which is essential for the retention of maternal components for early embryo development [1]. The oocyte polarization experiences several steps including spindle migration, spindle anchoring and cortical reorganization [2]. This series of oocyte polarization processes are controlled by microtubule and microfilament cytoskeletons [2], [3]. After germinal vesicle breakdown (GVBD), the centrally-formed spindle migrates to the cortex of the oocyte in an actin-dependent manner. In addition, microfilaments are enriched to form an actin cap, microvilli are lost in the region overlaying the spindle, and cortical granules (CGs) are redistributed to form a CG-free domain (CGFD) [4]–[6]. All these processes are called cortical reorganization and polarization. After cortical reorganization, a small polar body is extruded, leaving a highly polarized oocyte. Different from common ligand-mediated cell polarity, the formation of oocyte polarity and cortical reorganization is independent of any external ligand and the signal is intrinsic to the oocyte [7]. Meanwhile, meiotic spindles in oocytes have no centrosome, which indicates that specialized mechanisms may be responsible for the off-centre positioning of the spindles. Until now, the molecular details of oocyte polarization have not been completely understood.

Actin is shown to play key roles during this process [5]. Actin nucleation is driven by actin binding proteins that are regulated by at least three distinct biochemical mechanisms [8]. These nucleation factors include formin family proteins [9], proteins harboring tandem actin monomer (G-actin) binding surfaces in variable numbers [10] and the Arp2/3 complex with its activators [11]–[13]. Arp2/3 complex, the first factor shown to drive actin filament nucleation, consists of Arp2, Arp3 and five other subunits; Arpc1 to Arpc5 [12], [14]. Since its discovery, the Arp2/3 complex has been shown to be involved in a wide range of cellular processes, from migration and endocytosis to trafficking and cell–cell communication [14]. In many species, inhibition of the activity of the complex by RNAi or inhibitory antibodies induces the disruption of cell migration and adhesion [15]–[17], endocytosis [18], [19], and the establishment of cell polarity during mitosis [14], [20]. Recent works by others and us have shown that Arp2/3 complex is essential for the maintenance of meiotic spindle position in mouse oocytes [21], [22].

Arp2/3 complex is activated by a class of proteins termed nucleation-promoting factors (NPFs), including WASP [23], N-WASP [24], [25], WAVE1 [26], [27], WAVE2 [28], [29], WAVE3, WASH [30], WHAMM [31], Abp1 [32], Pan1, Cortactin [33]–[35] and JMY [36]. WAVE2, a critical actin nucleation regulator, is widely expressed in mammals and is a member of the WASP family [37], [38]. WAVE2 consists of three independent domains at the C-terminus: the verprolin homology (V), central (C) and acidic (A) regions. These domains can bind and activate the Arp2/3 complex, which is directly involved in actin nucleation [39], [40]. JMY is originally identified as a cofactor of the transcriptional regulator p300/CBP which augments the p53 response [41]. JMY contains a series of WH2 domains that facilitate actin nucleation, and the isolated C-terminus of JMY stimulates Arp2/3-dependent actin assembly as potently as neural-Wiskott-Aldrich syndrome protein-WH2, connector, acidic (N-WASP-WWCA) [42]. Our recent work shows that WAVE2 and JMY are involved in oocyte polarization during meiotic maturation [42], [43]. Meiotic spindle stability, peripheral positioning and polar body emission are disturbed after knock down of WAVE2 in mouse oocytes [43]. And JMY RNAi disrupts the spindle migration, asymmetric division and cytokinesis during mouse oocyte maturation [44].

Although roles of NPFs and ARP2/3 in mouse oocyte have been separately uncovered, the signaling pathway leading to actin nucleation during meiosis is still unclear. Since WAVE2, JMY and ARP2/3 are involved in oocyte asymmetric division, their similar roles make us to investigate the relationship between NPFs and ARP2/3 during meiosis. In the present study, we injected the siRNA of these molecules into mouse oocytes and showed that knock down of NPFs caused the disruption of ARP2/3 complex localization in oocyte meiosis. Our results indicated that NPFs showed conserved roles and were the upstream regulators of ARP2/3 in oocyte meiosis.

## Results

### JMY RNAi disrupts the localization of ARP2/3 complex in mouse oocyte

**Figure 1:**
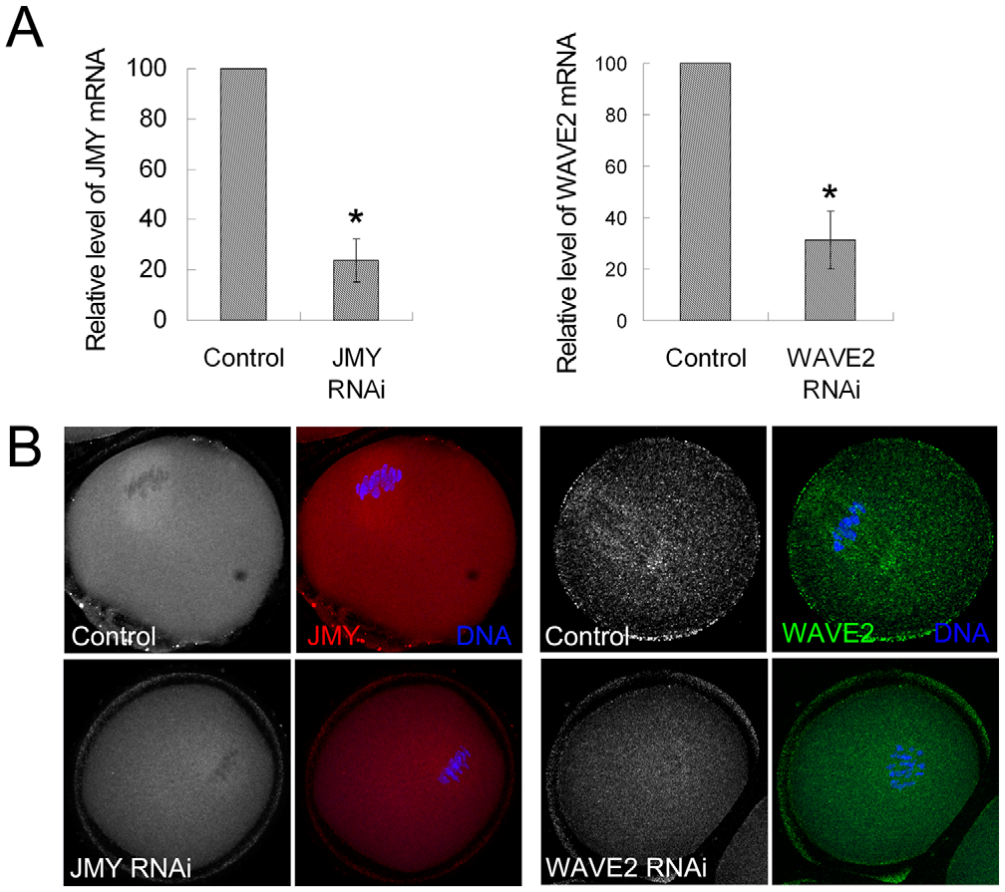
Efficiency of JMY and WAVE2 RNAi in mouse oocyte. (**A**) JMY and WAVE2 mRNA level after JMY and WAVE2 siRNA injection. *, significantly different (p<0.05). (**B**) Immunofluorescence staining of JMY and WAVE2 in the oocytes after JMY and WAVE2 RNAi. In the control group, JMY localized at the spindle and WAVE2 accumulated around the spindle, whilst in the RNAi group, JMY and WAVE2 were barely expressed.

**Figure 2:**
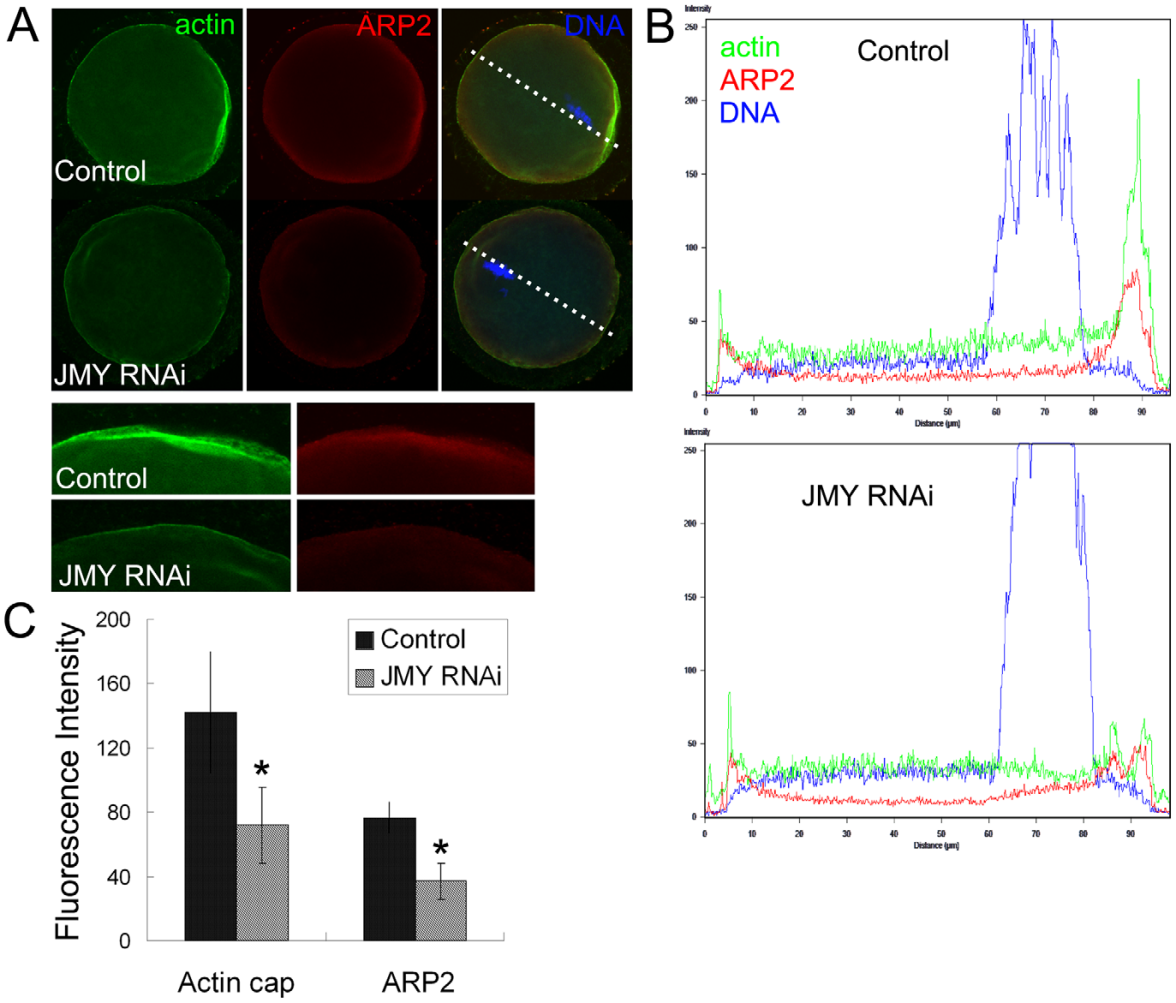
Expression and localization of actin and ARP2 after the injection of JMY siRNA. (**A**) Localization of actin and ARP2 in oocytes by immunofluorescence staining after JMY RNAi. The expression of actin and ARP2 was significantly weaker in the JMY RNAi group. Green, actin; red, ARP2; blue, chromatin. (**B**) Fluorescence intensity curve of dotted line area in oocytes. The fluorescence intensity of actin and ARP2 was significantly weakened in the JMY RNAi group. (**C**) The average fluorescence intensity of actin and ARP2 in mouse oocytes. *, significantly different (p<0.05).

To investigate the effect of JMY on ARP2/3 in mouse oocytes, we used JMY siRNA injection to down-regulate the expression of JMY, and successfully depressed the mRNA level of JMY (23.7±8.6% versus 100%) (Fig. 1A). Immunofluorescent staining was adopted to examine the expression of JMY in oocytes. As shown in Fig. 1B, in the control group JMY accumulated at the spindle of the oocytes and in the cytoplasm. Compared to the control group, the protein expression of JMY in the RNAi group was significantly decreased. Then we examined the protein expression and localization of actin and ARP2 in mouse oocytes. As shown in Fig. 2A, an actin cap formed at the area where the chromosomes were closed to in the control group and ARP2 also accumulated at the actin cap area of the oocytes and the cytoplasm, whilst no actin cap formed and no ARP2 accumulated at this area in the JMY RNAi group. To confirm this, we examined the fluorescence intensity curve of oocytes. As shown in Fig. 2B, the intensity of actin and ARP2 at the dotted line area was significantly weakened in the JMY RNAi group compared to the control group. We also examined the average fluorescence intensity of the oocytes. As shown in Fig. 2C, the fluorescence intensity of actin in the control group (142.2±37.5) was significantly higher compared to that in the JMY RNAi group (71.9±23.5), while the fluorescence intensity of ARP2 in the control group (76.3±9.4) was also significantly higher compared to that of the JMY RNAi group (37.1±11.3), which further confirmed that the inhibition of JMY disrupted the localization of ARP2/3 complex in oocytes.

### WAVE2 RNAi causes disruption of ARP2/3 complex localization in mouse oocyte

**Figure 3:**
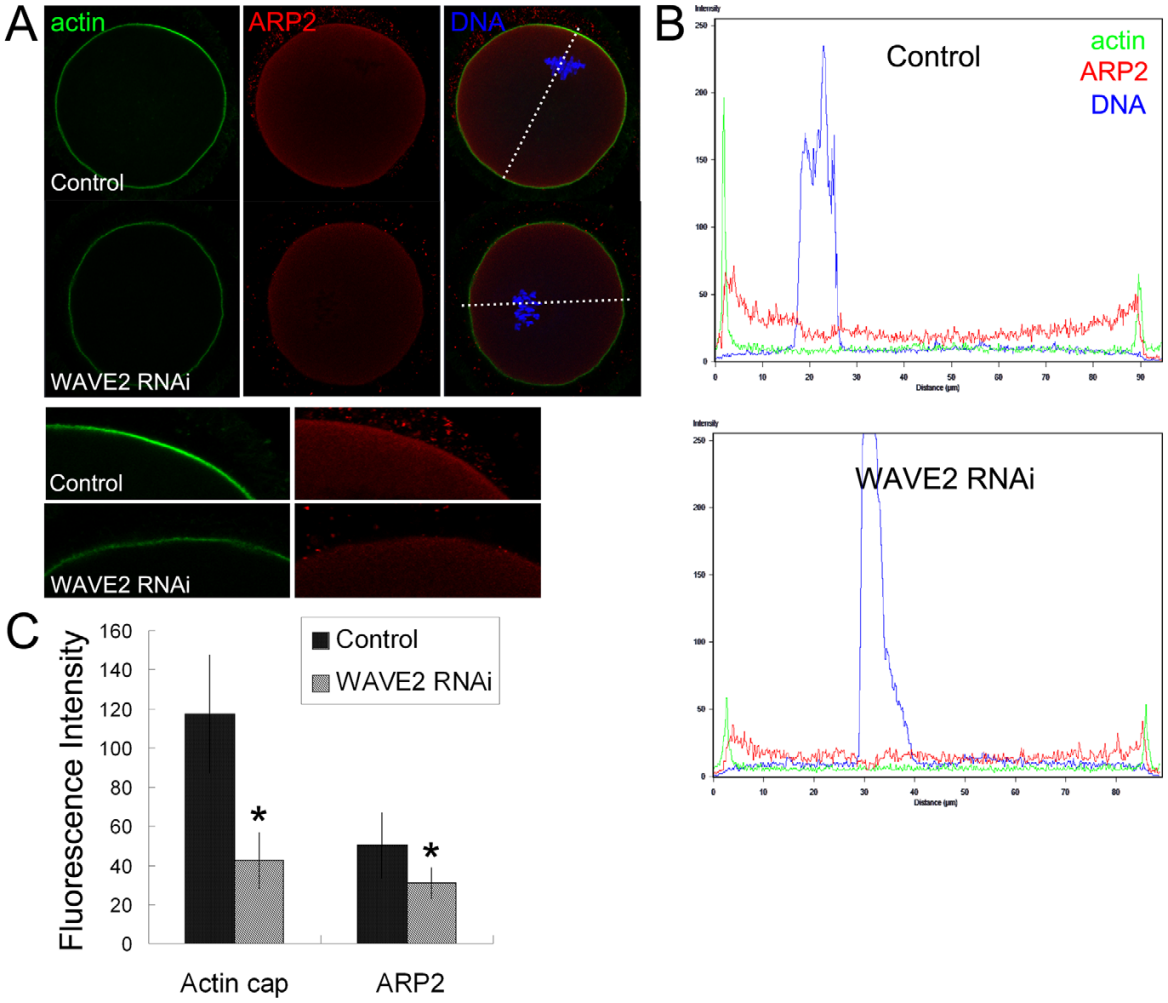
Expression and localization of actin and ARP2 after the injection of WAVE2 siRNA. (**A**) Localization of actin and ARP2 in oocytes by immunofluorescence staining after WAVE2 RNAi. The expression of actin and ARP2 was significantly weaker in the WAVE2 RNAi group. Green, actin; red, ARP2; blue, chromatin. (**B**) Fluorescence intensity curve of dotted line area. The fluorescence intensity of actin and ARP2 was weakened in the WAVE2 RNAi group. (**C**) The average fluorescence intensity of actin and ARP2 in oocytes. *, significantly different (p<0.05).

Analogously, to explore the effects of WAVE2 on ARP2/3 complex in oocytes, WAVE2 siRNA injection was used to down-regulate the expression of WAVE2, which successfully depressed the mRNA level of WAVE2 (31.3±11.2% versus 100%) (Fig. 1A). We also used immunofluorescent staining to examine the protein localization in the oocytes, and the results showed that WAVE2 accumulated around the spindle during spindle migration of late MI stage in the control group (Fig. 1B). However, WAVE2 was barely expressed in the WAVE2 RNAi group. Then we examined the protein expression and localization of actin and ARP2 in mouse oocytes. As shown in Fig. 3A, an actin cap formed and ARP2 accumulated at the cortex of the oocytes in the control group, but after WAVE2 siRNA injection, no actin cap was observed and no ARP2 accumulated. Furthermore, the fluorescence intensity curve of dotted line area also indicated that the fluorescence intensity of actin and ARP2 in the control group was significant higher than that in the WAVE2 RNAi group (Fig. 3B). Measurement of actin cap-ﬂuorescence intensity revealed that actin expression was decreased in the WAVE2 RNAi group compared to the control group (42.5±14.4 vs. 117.4±30.2). Similarly, the expression of ARP2 was also decreased in the WAVE2 RNAi group (30.9±7.8 vs. 50.2±16.7) (Fig. 3C). These results indicated that WAVE2 is essential for the localization of ARP2/3 complex in oocytes.

### Arp2/3 complex is not required for the localization of JMY and WAVE2 in mouse oocyte

**Figure 4:**
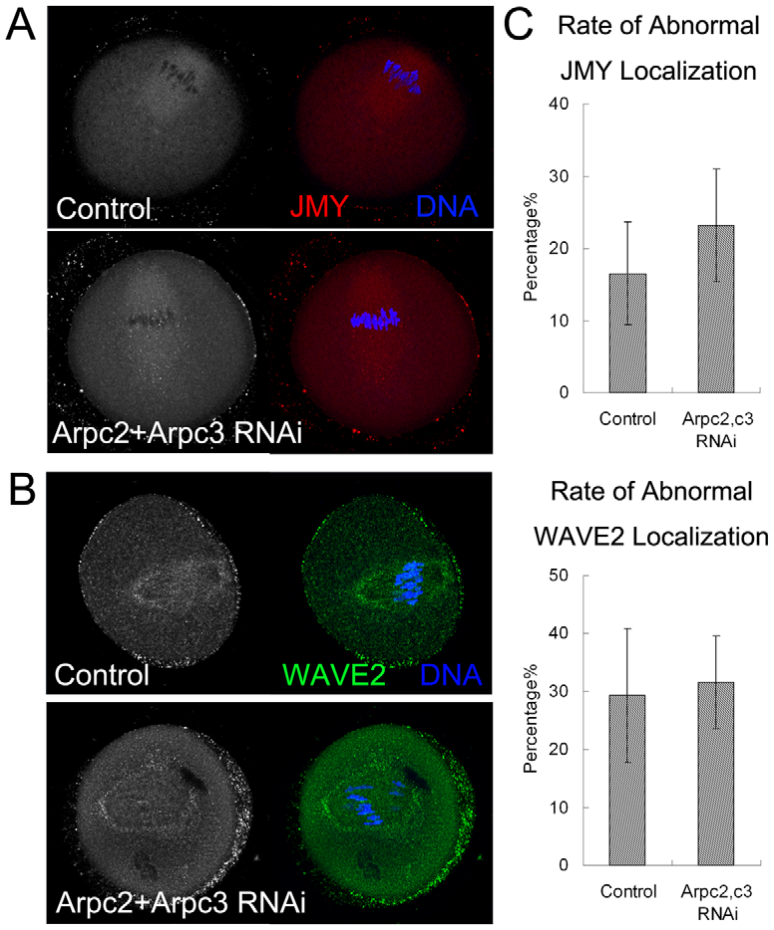
Expression and localization of JMY and WAVE2 after Arpc2 and Arpc3 RNAi. (**A**) Localization of JMY in oocytes by immunofluorescence staining after Arpc2 and Arpc3 RNAi. JMY expressed normally in both control and Arpc2 and Arpc3 RNAi group. (**B**) Localization of WAVE2 in oocytes by immunofluorescence staining after Arpc2 and Arpc3 RNAi. WAVE2 expressed normally in both control and Arpc2 and Arpc3 RNAi group. (**C**) Abnormal rate of JMY and WAVE2 localization in mouse oocytes after Arpc2 and Arpc3 RNAi.

To further confirm the relationship between NPFs and ARP2/3 complex, we knocked down ARP2/3 complex by Arpc2 and Arpc3 siRNA injection, and the mRNA levels were significantly decreased [22]. After the expression of ARP2/3 complex was decreased, we examined the expression and localization of JMY and WAVE2 in oocytes by immunofluorescent staining. As shown in Fig. 4A, JMY accumulated at the spindle in the control group, whilst there was no change for the localization of JMY in the Arpc2 and Arpc3 RNAi group, indicating that the inhibition of ARP2/3 complex had no effect on the localization of JMY in oocytes. Similarly, in both control and Arpc2 and Arpc3 RNAi group, WAVE2 expressed normally and accumulated around the spindle in the oocytes, which also revealed that ARP2/3 complex was not a regulator of WAVE2 (Fig. 4B).

To confirm this, the rate of abnormal JMY and WAVE2 localization was analyzed. The rate of abnormal JMY localization was no different for the Arpc2 and Arpc3 RNAi group compared with the control group (23.2%±7.8% vs. 16.5%±7.1%, n = 26, P>0.1) (Fig. 4C). Likewise, the rate of abnormal WAVE2 localization was also not different between the Arpc2 and Arpc3 RNAi group and the control group (31.6%±8% vs. 29.3%±11.6%, n = 32, P>0.1) (Fig. 4C). These results further demonstrated that ARP2/3 complex was not the upstream regulator of JMY and WAVE2.

## Discussion

In the present study, we investigated the relationship between NPFs and ARP2/3 complex during mouse oocyte meiotic maturation. By the knock down of JMY, WAVE2 and Arp2/3 complex, our results revealed that the NPFs JMY and WAVE2 were the upstream regulators of Arp2/3 complex in asymmetric mouse oocyte division.

JMY is a newly identified nucleation promoting factor. In our previous study, JMY has been demonstrated to be involved in spindle migration, asymmetric cell division, and cytokinesis in mouse oocytes [44], but the mechanisms how JMY affects these series of processes are unknown. Previous work showed that Arp2/3 complex might be the target of JMY for the actin nucleation. The isolated C-terminus of JMY (WWLWCA; L refers to a short linker sequence discussed below) stimulated Arp2/3-dependent actin assembly as potently as N-WASP-WWCA [8]. In vitro, JMY-WWWCA promotes actin-based motility by an Arp2/3 complex–dependent mechanism, and full-length JMY nucleates actin filaments and activates the Arp2/3 complex [45]. In the current study, we attempted to find whether JMY was an upstream regulator of ARP2/3 complex in the unique mammalian meiotic oocyte model. Since ARP2/3 complex was proved to be involved into asymmetric division and cytokinesis in mouse oocytes [22]. The results showed that Arp2/3 complex failed to accumulate to the cortex area of oocyte after JMY RNAi, indicating that the regulatory roles of JMY on Arp2/3 complex, and that the proper localization of ARP2/3 complex in mouse oocyte requires the expression of JMY. Therefore, a JMY-Arp2/3 complex-actin signaling pathway was set up for the regulation of mammalian oocyte asymmetric division.

WAVE2, a WASP family member, is a traditional NPF binds to Arp2/3 complex and active it in mitosis [12], [14]. It also stimulates localized actin polymerization through its interaction with the Arp2/3 complex [46], [47]. It was shown in previous works that *C. trachomatis* activated Rac and promoted its interaction with WAVE2 to activate the Arp2/3 complex, resulting in the induction of actin cytoskeletal rearrangements required for chlamydial invasion [48]. Recently, the roles of WAVE2 in mouse oocyte was also revealed by us, and it was shown that WAVE2 might regulate p42/44 MAPK for the meiotic spindle formation [43]. However, it is still unclear how WAVE2 works for the meiotic spindle migration and asymmetric division in mouse oocyte. Our results showed that Arp2/3 complex might be the downstream molecule of WAVE2, since the expression of ARP2 decreased after WAVE2 RNAi. Therefore, in mammalian oocytes WAVE2 may regulate Arp2/3 complex to active actin assembly for the meiotic spindle migration and the asymmetry. Our data suggest that WAVE2 is an upstream regulator of ARP2/3 complex, and a WAVE2-Arp2/3-actin signaling pathway was set up for the regulation of mammalian oocyte asymmetric division.

To examine how Arp2/3 complex interacts with NPFs, we also examined the expression of JMY and WAVE2 after the depletion of Arp2/3 complex, and the results showed that there was no effect on the localization of these NPFs. Therefore, the regulatory mechanism for NPFs on Arp2/3 complex was not mutual in mouse oocytes; instead, NPFs functioned as the upstream molecules of Arp2/3 complex. Our results provide direct evidence of the regulation of NPFs on ARP2/3 complex during mouse oocytes maturation.

In conclusion, our results suggest that the inhibition of JMY and WAVE2 causes disruption of ARP2/3 complex localization in meiosis, indicating that NPFs JMY and WAVE2 are the upstream regulators of ARP2/3 complex in mouse oocytes.

## Materials and Methods

### Ethic statement

Animal care and use were conducted in accordance with the Animal Research Institute Committee guidelines of Nanjing Agricultural University, China. Mice were housed in a temperature-controlled room with proper darkness-light cycles, fed with a regular diet, and maintained under the care of the Laboratory Animal Unit, Nanjing Agricultural University, China. The mice were killed by cervical dislocation. This study was specifically approved by the Committee of Animal Research Institute, Nanjing Agricultural University, China.

### Antibodies and chemicals

Goat polyclonal anti-JMY antibody and rabbit polyclonal anti-WAVE2 antibody were purchased from Santa Cruz (Santa Cruz, CA), whilst mouse monoclonal anti-ARP2 antibody was obtained from Abcam (Cambridge, UK). Phalloidin-TRITC, Lectin-FITC and mouse monoclonal anti-α-tubulin-FITC antibody was purchased from Sigma (St Louis, MO). Alexa Fluor 488, 568 antibodies were obtained from Invitrogen (Carlsbad, CA).

### Oocyte collection and culture

Germinal vesicle-intact oocytes were collected from ovaries of 6- to 8-week-old ICR mice and cultured in M16 medium (Sigma, MO) under paraffin oil at 37°C 5%CO_2_. After a range of times in culture, oocytes were collected for immunostaining, real time RT-PCR and microinjection.

### Microinjection of siRNA

Approximately 5–10 pl JMY siRNA (Sigma) was microinjected into the cytoplasm of a fully grown GV oocyte using a Eppendorf FemtoJet (Eppendorf AG, Hamburg, Germany) with a Nikon Diaphot ECLIPSE TE300 inverted microscope (Nikon UK Ltd., Kingston upon Thames, Surrey, UK) equipped with a Narishige MM0-202N hydraulic three-dimensional micromanipulator (Narishige Inc., Sea Cliff, NY). The oocytes were then cultured in M16 medium contained 5 μM milrinone for 24 h after injection. After culture, the oocytes were washed five times in fresh M16 medium, for 2 min each time, and then transferred to fresh M16 medium and cultured under paraffin oil at 37°C in an atmosphere of 5% CO_2_ in air. For the control oocytes, 5–10 pl of negative control siRNA were microinjected. The spindle, actin cap phenotypes and chromosome localization were examined by confocal microscopy (Zeiss LSM 710 META, Germany). For WAVE2, Arpc2 and Arpc3 siRNA injection, the same method was adopted as above.

### Real-time quantitative PCR analysis

Analysis of JMY and WAVE2 gene expression was measured by real-time quantitative PCR and the ΔΔC_T_ method. We used a Dynabead mRNA DIRECT kit (Invitrogen Dynal AS, Norway) to extract the total RNA from 50 oocytes. The first strand cDNA was generated with a cDNA synthesis kit (Takara), using Oligo(dT)12–18 primers (Invitrogen). JMY cDNA fragment was amplified using the following primers: F, TTC AAA TTA CAA GCC GTG CAC CCG; R, AGC TGC CTT CTG GAC CTT TAC TGA, while WAVE2 cDNA fragments were amplified using the following primers: F, AAC TCC ATG CTG TGC ATG TTT CCC; R, TCT ATT TGG AAG GAC CAC TGC CCT.

The DyNAmo HS SYBR Green qPCR kit (FINNZYMES) was used with a DNA Engine OPTICON 2 Continuous Fluorescence Detector (MJ Research) under the following conditions: 95°C for 10 sec, and 38 cycles of 95°C for 5 sec and 59°C for 32 sec.

### Confocal microscopy

The protocol was basically the same as described in our previous work [44]. For immunostaining, oocytes were fixed in 4% paraformaldehyde in PBS for 30 min at room temperature and then transferred to membrane permeabilization solution (0.5% Triton X-100) for 20 min. After 1 h in blocking buffer (1% BSA-supplemented PBS), oocytes were incubated overnight at 4°C or for 4 h at room temperature with 1∶100 goat anti-JMY (1∶50 rabbit anti-WAVE2, 1∶200 mouse anti-ARP2) antibody. After three washes in washing buffer (0.1% Tween 20 and 0.01% Triton X-100 in PBS), the oocytes were labeled with 1∶100 Alexa Fluor rabbit-anti-goat 488 IgG or donkey anti-goat 568 IgG for 1 h at room temperature. The samples were co-stained with Hoechst 33342 (10 μg/ml in PBS) for 10 min and then mounted on glass slides and examined with a confocal laser-scanning microscope (Zeiss LSM 710 META, Germany). At least 20 oocytes were selected for examination for each group.

### Statistical analysis

Three replicates were performed for each experiment. Statistical analyses were conducted using an analysis of variance (ANOVA) and differences between treatment groups were evaluated with Duncan's multiple comparison tests. Data were expressed as mean ± SEM and p<0.05 was considered significant. Fluorescence intensity statistics were examined by Image J (NIH) software and 10 oocytes were analyzed for each experiment.
